# TRviz: a Python library for decomposing and visualizing tandem repeat sequences

**DOI:** 10.1093/bioadv/vbad058

**Published:** 2023-04-26

**Authors:** Jonghun Park, Eli Kaufman, Paul N Valdmanis, Vineet Bafna

**Affiliations:** Department of Computer Science & Engineering, University of California, San Diego, La Jolla, CA 92093, USA; Division of Medical Genetics, University of Washington School of Medicine, Seattle, WA 98195, USA; Division of Medical Genetics, University of Washington School of Medicine, Seattle, WA 98195, USA; Department of Computer Science & Engineering, University of California, San Diego, La Jolla, CA 92093, USA

## Abstract

**Summary:**

TRviz is an open-source Python library for decomposing, encoding, aligning and visualizing tandem repeat (TR) sequences. TRviz takes a collection of alleles (TR containing sequences) and one or more motifs as input and generates a plot showing the motif composition of the TR sequences.

**Availability and implementation:**

TRviz is an open-source Python library and freely available at https://github.com/Jong-hun-Park/trviz. Detailed documentation is available at https://trviz.readthedocs.io.

**Supplementary information:**

Supplementary data are available at *Bioinformatics Advances* online.

## 1 Introduction

Tandem repeats (TRs) are a type of DNA sequence in which a motif is repeated multiple times in a consecutive manner, with the possibility of variations among the repeating motif. A substantial portion of the human genome is comprised of TRs, which often have large motif lengths. TRs are often highly polymorphic in a population, contributing to significant genetic variation and mediating gene expression (Bakhtiari *et al.*, 2021). They have also been associated with more than 50 human traits, including genetic disease (Mitra *et al.*, 2021; Mukamel *et al.*, 2021).

Genetic variation in TRs can change the copy number of motifs (or repeat units), resulting in changed allele length. Many bioinformatics tools detect such length variation (Bakhtiari *et al.*, 2021; Dolzhenko *et al.*, 2019; Mousavi *et al.*, 2019). TRs, especially variable number tandem repeats or VNTRs with long motifs, can harbor other types of variation, including substitutions and small indels resulting in *imperfect* tandem repeats comprised of multiple motifs.

Due to the complex variation patterns, it can be challenging to associate specific motifs or alleles with a phenotype. For example, unique VNTR motifs have been reported to be causal for phenotypes (Lu *et al.*, 2023; Song *et al.*, 2018), however, detecting such motifs can be difficult when they appear in a highly complex background. In such cases, a visual display of TR motif composition in multiple individuals, similar to multiple sequence alignments, can be an effective tool to quickly highlight the causal motif. This approach can be especially useful in exploring the complexity and impact of variation in TR regions. The visualization can generate specific hypotheses that can subsequently be assessed with appropriate statistical tests.

While there were visual displays to show the TR polymorphisms, those efforts were either manually generated (Course *et al.*, 2021), or alternatively used fixed k-mer motifs, which are not suitable for variable length motifs (Song *et al.*, 2018; Sulovari *et al.*, 2019). This is in part due to the complexity of resolving TR alleles and identifying and comparing the structure of TR sequences.

Here, we present TRviz, an open-source python library for visualizing TR sequences. TRviz provides modules for decomposing, encoding, aligning and visualizing TR alleles. It may be used to analyse any type of tandem repeats.

## 2 Features and methods

**Fig. 1.**
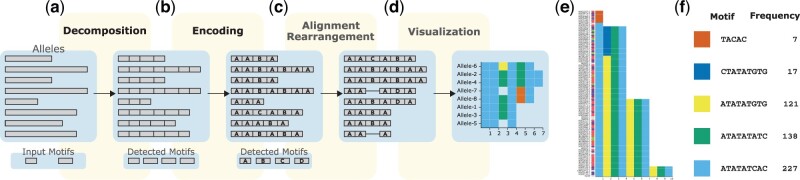
An overview of TRviz. (**a**) Decomposition: given tandem repeat (TR) sequences and a set of motifs, the module outputs decomposed sequences comprised of a series of detected motifs. (**b**) Encoding: each distinct motif is labeled as a symbol. (**c**) Alignment and rearrangement: given the encoded TR alleles, it performs multiple sequence alignment and rearranges the order. (**d**) Visualization: this module generates a plot showing the TR motif structure and composition. For each distinct motif, a distinct color is assigned. For example, the motif colored in red could indicate a disease-associated motif. (**e**) A plot generated by TRviz for a TR in *MALRD1* (chr10:1917995-19172084). Rows represent the decomposed TR alleles extracted from human genome assemblies. The sample names are labeled on the *y*-axis. Next to the names, color-coded labels can be used to annotate the group membership. In this example, the super population code was used. (**f**) The mapping between motifs and actual sequences detected in the TR alleles and their frequency in (e)

TRviz generates a plot in four steps, each available as a standalone module: decomposition, encoding, alignment and rearrangement and visualization (Fig. 1a–d). The output of each module is the input of the following module. In the next sections, we describe each module in detail.

*a. Decomposition of tandem repeat sequences*. The decomposition module takes a collection of *alleles* (TR containing sequences) and one or more motifs. It automatically generates an ‘optimum’ decomposition of each allele into motifs (Fig. 1a).

To clarify the decomposition problem, we define Σ={A,C,G,T} and $\Sigma^{+}$ as a set of sequences composed of the symbols from Σ. For $x,y\in \Sigma^{+}$, let d(x,y) denote a score function between *x* and *y*. Let $M\subseteq \Sigma^{+}$ represent a collection of input motifs. Given a TR allele $x\in \Sigma^{+}$, the decomposition problem is to *optimally parse x*, by partitioning *x* into substrings $x^{\left(1\right)},x^{\left(2\right)},x^{\left(3\right)},\ldots$ such that is maximized. In this optimum parse, we say that the substring $x^{\left(i\right)}$ is ‘aligned’ to motif *m*. If $x^{\left(i\right)}$ is aligned to m∈M in the optimum parse, and $x^{\left(i\right)}=m$, then $x^{\left(i\right)}$ denotes an input motif; otherwise, $x^{\left(i\right)}$ represents a new motif.


(1) $$D(x,M)=\sum_{i}\underset{m\in M}{\max}d(x^{\left(i\right)},m)$$

We solve the optimum parse problem by using dynamic programming. Let *D*[*i*, *m*, *j*] denote the maximum score of aligning the prefix of the query sequence *x*[1.*i*] to a collection of motifs *M*, where the *i*th character of the query sequence is matched to the *j*th character of motif *m*. Solving the recurrence using dynamic programming (see Supplementary Information) provides the optimum score as where |x| and |m| denote the length of the query and motif, respectively. We employed the edit distance as the scoring function d(x,y), assigning a score of 5 for matches, –4 for mismatches and indels by default. The final decomposition is constructed using standard backtracking methods.


(2) $$\underset{m}{\max}\{D[|x|,m,|m|]\},$$

The method is similar to the ‘string decomposer’ algorithm (Dvorkina *et al.*, 2020), with some differences, including the possible decomposition into motifs not given by the initial motif set. This is useful for TRs with previously unknown, complex motif structures. As a simple example, the following code snippet uses a novel motif ACCTTTG, to complete the decomposition.

>>> from trviz.decomposer import Decomposer

>>> tr_decomposer = Decomposer()

>>> tr_sequence = "ACCTTGACCTTGACCTTTG"

>>> motifs = ["ACCTTG"]

>>> tr_decomposer.decompose(tr_sequence, motifs)

>>> ["ACCTTG", "ACCTTG", "ACCTTTG"]

*b. Encoding decomposed motifs*. Similarly, we define Γ to denote a second set of symbols, representing encoded motifs, and $\Gamma^{+}$ to be a set of sequences composed of symbols from given Γ, representing encoded TR alleles. Given decomposed TR alleles, each represented as a list of motifs, the encoding module sets a mapping function from motifs to symbols, $e:\Sigma^{+}\mapsto \Gamma$, and converts the decomposed allele into a sequence of symbols $a\in \Gamma^{+}$ using the mapping function and output the mapping as a text file (Fig. 1b). A maximum of 90 distinct symbols (motifs) are allowed, with the option of assigning a special symbol to encode rare private motifs. The frequency threshold for private motifs can be set by users, or an ‘auto’ option can be used to maximize the number of symbols.

*c. Alignment and rearrangement of encoded motifs*. To align the encoded alleles, TRviz performs multiple sequence alignment using MAFFT (Katoh and Standley, 2013) and outputs the aligned sequences (Fig. 1c). We use a customized scoring matrix to align the symbols, not nucleotides (see Supplementary Information). For every symbol pair, we define a mismatch score based on the similarity between the corresponding pair of motifs. The scoring is optimized to avoid introducing gaps between two quite similar motifs. After the alignment step, TRviz rearranges the aligned sequences (Fig. 1c) to bring similar alleles together. By default, TRviz performs hierarchical clustering and rearranges the alleles based on the clusters. We support other rearrangement methods for different visualization goals, including sorting by motif counts and manual rearrangement.

*d. Visualization*. The visualization module generates a plot showing the motif composition of the alleles given the aligned and encoded alleles (Fig. 1d). The encoded alleles are color-coded, and a distinct color is automatically assigned to each symbol, including grey (by default) for private motifs. In Figure 1e, we visualized a collection of 96 alleles extracted from human assemblies generated by the Human Pangenome Reference Consortium (HPRC) at the chr10:19171995-19172084 VNTR locus. Each row represents a distinct allele sampled from a specific super-population. The participating motifs and their color codes are shown in Figure 1f.

## 3 Results

**Fig. 2.**
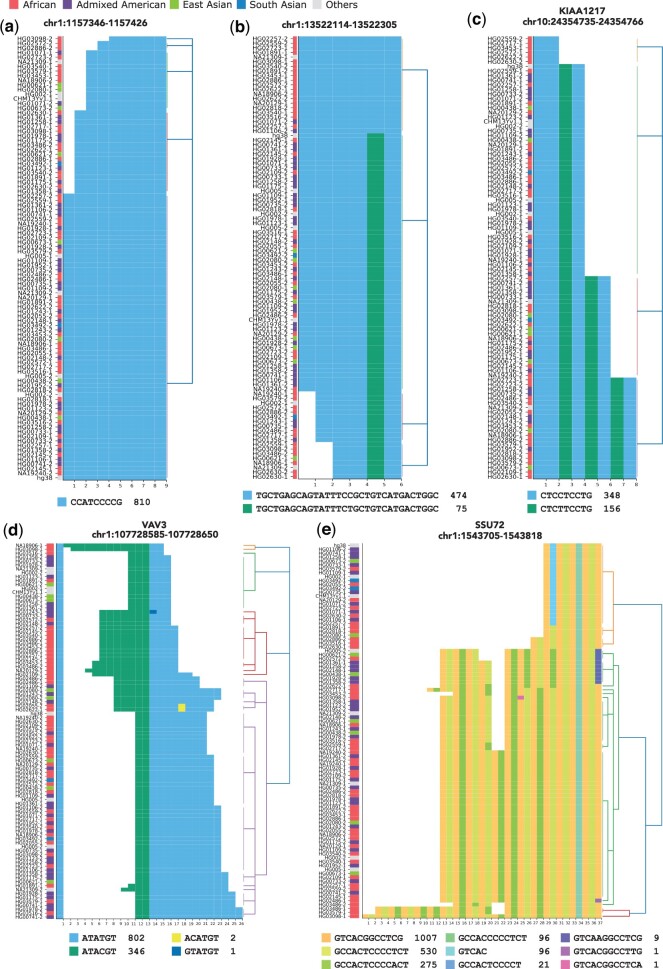
Human tandem repeat polymorphisms. (**a**) A ‘pure’ variable number tandem repeat (VNTR) with a single motif. The color codes for the super population are shown at the top of the figures. (**b**) and (**c**) VNTRs with two detected motifs that differ by a single nucleotide. It should be noted that some alleles only carry a single motif and are mostly found in African population (marked in red). (**d**) A VNTR composed of two variable motifs (in sky blue and green) and two low-frequency motifs (in yellow and blue). Note that some alleles share the same number of motifs but have different motif compositions. (**e**) Repeat expansion with a set of motifs. The expanded allele pattern reveals a fusion of two separate motifs. All alleles in this figure were extracted from the human assemblies generated by the Human Pangenome Reference Consortium

To demonstrate the applicability of the method, we visualized various TR loci using the alleles extracted from human assemblies generated by HPRC (Fig. 2 and Supplementary Fig. S1), which have provided insights into the complex variation in motif structure and allele lengths.

Figure 2a illustrates one of the simpler cases: a TR locus composed of a single, 9-bp-long motif. In such a TR composed of exact repeats of a single motif, variations in the number of motifs can be identified by using TR genotypers, such as adVNTR (Bakhtiari *et al.*, 2021), which mainly look at length differences in alleles. However, these methods may not fully capture variations occurring within the motifs, for example in Figure 2b and c. In each of these examples, the VNTRs have two motifs that are one edit distance apart. For example, in Figure 2b, the majority of alleles have the same number of motifs but with different motif compositions; the difference between HG01106 and HG02145 would not be captured by most VNTR genotyping tools. Interestingly, the alleles containing a single motif are primarily found within the African population. While visually apparent by looking at the population code in Figure 2b, this observation could be easily missed by tools analyzing the data statistically.

Figure 2d presents an intriguing case where the number of two quite similar motifs is highly variable, even though the total length of the TR may not vary. For example, HG01109-2 and HG03486-2 have identical lengths, but different compositions. Furthermore, TRviz detected two private motifs represented in only a few alleles. These detected rare motifs can be used to generate a hypothesis, which would not be captured by most VNTR genotyping tools.

Figure 2e depicts a complex VNTR region located in the intron of *SSU72* gene. About 78% of the alleles have longer lengths compared to hg38 and T2T reference alleles. The expanded alleles show a striking pattern carrying a group of motifs together when expanding. It is noteworthy that not all motifs have the same length, thus a fixed k-mer-based decomposition method would not be suitable for detecting the pattern correctly, and methods testing hypotheses based on lengths could miss some of the higher order patterns.

Finally, we visualized the VNTR in *SORL1* gene (chr11:121480539-121482037) of 110 alleles including 6 alleles from three different non-human primates; orangutan, gorilla and chimpanzee, which had been previously reported to have a human-specific expansion (Course *et al.*, 2021). Our tool confirmed this expansion (Supplementary Fig. S1) but also identified human alleles (largely African) with a novel deletion, or a non-expansion of motifs along with a different pattern in the fourth motif.

The runtime is dominated by the decomposition and alignment steps. Decomposition runtime is dependent on the length and number of the TR alleles and input motifs. As an example, visualizing a TR with 300 alleles of average length 40 bp and a 10 bp motif took 16 secs on a single 3.8 GHz CPU. For a more complex example, visualizing 110 alleles of average length 1559 and 57 bp motifs took 30 s for a SORL1 VNTR shown in Supplementary Figure S1.

## 4 Discussion

Tandem repeats are the ‘hidden polymorphisms,’ that govern a large fraction of human genetic variation, and mediate both Mendelian and complex phenotypes. In recent years, many statistical tools have been developed for analyzing tandem repeats, and identifying previously hidden genetic factors involved in specific phenotypes (Bakhtiari *et al.*, 2021; Dolzhenko *et al.*, 2019; Mousavi *et al.*, 2019; Mukamel *et al.*, 2021). While successful, these methods typically focus on specific aspects of variation. For example, they may look for length differences, or more rarely, for single nucleotide variants within repeats. However, TR variation can take complex forms, relating to changes in the number of copies of different repeat units while preserving overall composition. In these cases, a visual exploration could be helpful in formulating a hypothesis, for which an appropriate statistical test could be devised. The development of such tools is technically challenging due to the complex patterns of variation.

TRviz is a software tool that provides a direct view of the complexity and evolution of a polymorphic repeat locus. Rather than work as a multiple alignment tool, it is motif-centric and clearly displays the order and number of different motifs. It is length agnostic, and can display motifs of different lengths. Thus, it can highlight subtle differences in TRs between groups of individuals, for example, subpopulations, closely related species, or even cases and controls for a phenotype mediated by variation in tandem repeats.

The notion of a motif is not well-defined. For example, a motif CTTACCGATT, could also be written as two distinct motifs CTTAC, and CGATT, which appear in tandem. The choice of one or two motifs is largely a matter of preference, but a parsimonious explanation is usually the preferred one. Thus, if they always appeared together in the same order, it would make sense to treat them as a single motif. Similarly, repeated occurrences of a motif (e.g. CTTAC-CTTAC-CTTAC) could easily be shifted by a few base-pairs (e.g. C-TTACC-TTACC-TTAC). Again, the choice is governed by unique sequences at the beginning and end of the tandem repeats, but different tools could make different choices. For these reasons, TRviz takes in a user-defined collection of motifs, and the TR boundaries as input. This allows a user to experiment with different motifs and visually explore the higher-order patterns. They could also compare the visualizations of two different motif structures to explore if groups of alleles are better discriminated by one choice. We note that other tools (e.g. Tandem Repeats Finder) can easily generate these inputs for the user if needed.

Another feature of TRviz is that complex repeats where two distinct TRs are together, or a TR nested within another TR can also be visualized simply by supplying appropriate motifs. However, we note that the visualization may be imperfect in some cases because of the high complexity of motif structure and the use of an off-the-shelf multiple alignment method. Improved understanding of TR evolution and better scoring functions will further improve visualizations, and how these complex polymorphisms mediate phenotypes.

## Supplementary Material

vbad058_Supplementary_Data

## Data Availability

There are no new data associated with this article. No new data were generated or analysed in support of this research.
