# Supplementary material for: TRviz: a Python library for decomposing and visualizing tandem repeat sequences
Source: Bioinform Adv. 2023 Apr 26;3(1):vbad058. doi: 10.1093/bioadv/vbad058 (PMC10166586; doi:10.1093/bioadv/vbad058)
Supplement: vbad058_Supplementary_Data [file vbad058_supplementary_data.pdf]

Supplemental Information

a

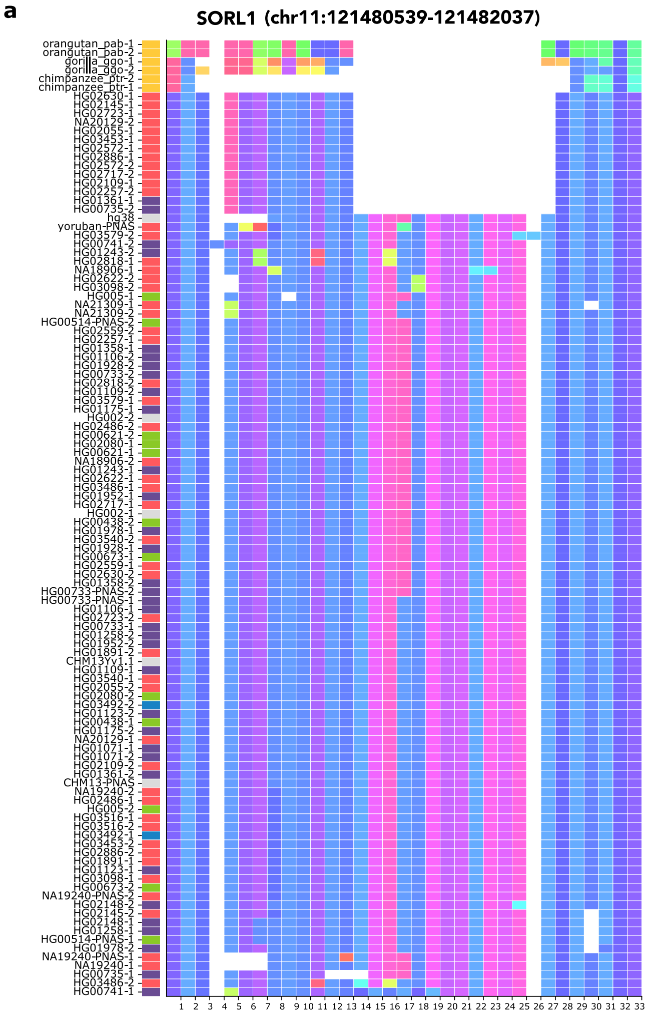

b

CATCTCCTCTTCCCCAGCTTCTTCCGTAGTGCACAGATACCTATAGGCAGGCTT	3
CATCTCTTCCTCCCCAGCTCCTCCCCTGGTGCACAGATACCTATAGGCAGGCTC	4
CATCTCCTCCTCCTCACCTCCTGCTGTGGTGCACAGATACCTATAGGCAGGCTT	4
CATCTCCTCCTCCCCAGCTCCTCCCCTAGAGCACAGATGTCTATAGGCAGGCTC	12
CATCTCCTCCTCCCCAGCTCCTCCCCTAGTGCATAGATACCTATAGGCAAGCTC	14
CATCTCCTCCTCCCCAGCTCCTCTCCTAGTGCACAGATACCTATAGGCAAGCTC	37
CATCTCTTCTTCCCCAGCTTCTTCCGTAGTGCACAGATACCTATAGGCAGGCTC	86
CTTCATCTCCCCAGCTCCTCCCCTAGTGCACAGATACCTATAGGCAGGTTC	88
CATCTCCTCCTCCCCAGCTCCTTCCCTAGTGCACAGATACCTATAGGCAGGCTT	89
CATCTCCTCCTCCCCAGCTCCTCCCCTAGTGCACAGATACCTATAAGCAAGCTC	89
CATCTCCTCCTCCCCAGCTCCTCCCCTAGTACACAGATACCTGTAGGCAGACTC	89
CATCTCCTCCTCCCCAGCTCCTCCCCTAGTGCACAGATACCTATAGGCAGG	90
CATCTCCTCCTCCCCAGCTCCTCTCCTAGTGCACAGATACCTATAGGCAGGCTT	90
CATCTCCTCCTCCCCAGCTTCTTCCATAGTGCACAGATACCTATAGGCAGGCTC	92
CATCTCCTCCTCCCCAGCTCCTCCCCTAGTGCACAGATACCTATCGGCAGGCTC	95
CATCTCTTCTTCCCCAGCTTCTTCCGTAGTGCACAGATACCTATAGGCAGGCTT	101
CATCTCCTCCTCCCCAGCTCCTCCCCTAGTGCACAGATACCAATAAGCAAGCTC	101
CATCTCTTCTTCCCCAGCTTCTTCCATAGTACACAGATGCCTATAGG	104
CATCTCCTCCTCCTCACCTCCTGCTGTGGTGCACAGATACCTATAGGCAGGCTC	104
CATCTCCTCCTCCCGAGCTCCTCCCCTAGTGCACAGATACCTATAGGCAGGCTC	105
CATCTCCTCCTCCCCAGCTCCTCCCCTAGTGCACAGATGTCTATAGGCAGGCTC	117
CATCTCCTCCTCCCCAGCTTCTCCCCTAGTGCACAGATACCTATAGGCAGGCTC	120
CATCTCCTCCTCCCCAGCTCCTCCCCTAGTACACAGATACCTATAGGCAGGCTT	202
CATCTCCTCCTCCCCAGCTCCTCCCCTAGTGCACAGATACCTATAGGCAGGCTC	289
CATCTCCTCCTCCCCAGCTCCTCCCCTAGTGCACAGATACCTATAGGCAAGCTC	341
CATCTCCTCCTCCCCAGCTCCTCCCCTAGTGCACAGATACCTATAGGCAGGCTT	583
CATCTCTTCTTCCCCAGCTCCTCCCCTAGTGCACAGATACCTATAAGCAAGCTC	1
CATCTCCTCCTCCCCAGCTCCTCCCCTAGTGCACAGATACCTATAGGCAGGTTC	1
CTTCATCTCCCCAGCTCCTCCCCTAGTGCACAGATACCTATAGGCAGGCTT	1
CATCTCCTCCTCCCCAGCTCCTTCCCTGGTGCACAGATACCTATAGGCAGGCTT	1
CATCTTCTCCTCCCCAGCTCCTCCCCTAGTGCACAGATACCTATAGGCAGGCTT	1
CTTCATCTCCCCAGCTCCTCCCCTAGTGCACAGATACCTACAGGCAGGTTC	1
CATCTCCTCCCCAGCTCCTCCCCTAGTGCACAGATACCTATAGGCAGGCTT	1
CATCTCTTCTTCCCCAGCTTCTTCCGTAGTACACAGATGCCTGTAGG	2
CACCTCCTCCTCCCCAGCTCCTCCCCTAGTGCACAGATACCTATAGGCAGGCTT	2
CATCTCCTCCTCCCCAGCTCCTCCCCTAGTGCACAGATACCTACAGGCAGGCTT	2
CATCTCTTCTTCCCCAGCTTCTTCCATAGTGCACAGATGCCTATAGG	2
CATCTCCTCCTCCCCAGCTCCTCCCTAGTGCACAGATACCTATAGGCAAGCTC	2
CATCTGTTGTTCCCCAGCTTCTTCCATAGTACACAGATGCATATAGG	2
CATCTCTTCTTCCCCAGCTCCTCCCCTAGTGCACAGATGTGTATAGGCAGGCTC	2
CATCTCCTTCCCAGCTCCTCCCCTAGAGCACAGATGTCTGTAGGCAGGCTC	2
CATCTCTTCTTCCCCAGCTTCTCCCCTAGTGCACAGATGTCTATAGGCAGGCTC	2
CATCTCTTCTTCCCCAGCCCCTCCCCTAGAGCACAGATGTCTATAGGCAGGCTC	2
CATCTCTTCTTCCCCAGCTCCTCCTCTAGAGCACAGATGTCTATAGGCAGGCTC	2
CATCTCTTCTTCCTCAGCTCCTCCCCTAGTGCACAGATGTCTATAGGCAGGCTC	2
CATCTCTTCTTCCCCAGCTCCTCCCCTAGAGCACAGATGTCTATAGGCAGGCTC	2
CATCTCCTCCTCCTCCTCACCTCCTGCCGTGGTGCATGGATGTCTATAGGCAGGCTC	2
CATCTCCTCCACCCCAGCTCCTCCCCTAGTGCACAGATACCTATCGGCAGGCTC	2
CATCTCCTCCTCCCCAGCTCCTTCCCTAGTGCACAGATACCTATAGGCAGGCTC	2
CATCTCCTCCTCCCCAGCTCCTCCCCTAGTGCACAGACACCTATAGGCAAGCTC	3
CATCTCCTCCTCCCCAGCTCCTCCCCTGGTGCACAGATACCTATAGGCAGGCTC	3
CATCTGTTCTTCCCCAGCTTCTTCCGTAGTGCACAGATACCTATAGGCAGGCTC	3
CATCTCCTCCTCCCCAGCTCCTCCCCTAGTGCACAGATACCATAGCAAGCTC	1
CATCTCTTCTTCCCAGCTCCTCCCCTAGTGCACAGATACCTATAGGCAGGCTC	1
CGTCTCCTCCTCCCCAGCTCCTCCCCTAGTGCACAGATACCTATAGGCAGGCTC	1
CATCTCCTCCTCCCCAGCTCCTCCCCTGGTTCACAGATACCTATAGGCAAGCTC	1
CATCTCTCCTCCCCAGCTCCTCCCCTAGTGCACAGATACCTATAGGCAGGCTC	1
CATCTCTTCTTCCCCAGCTCCTCCCCTAGTGCACAGATACCTATAGGCAGGCTT	1
CATCTCTTCTTCCCCAGCTTCCTCCATAGTGCACAGATACCTATAGGCAGGCTC	1
CATCTCTTCTCCCCAGCTCCTCCCCTAGTGCACAGATACCTATAGGCAGGCTC	1
CGTCTCCTCCTCCCCAGCTCCTCCCTAGTGCACAGATACCTATAGGCAGGCTC	1
CATCTCCTCCTCCCCAGCTCCTCCCCTGGTGCACAGATACCTATAGGCAGCTC	1
CATCTCCTCCTCCCCAGCTCCTCCCTAGTACACAGATACCTATAGGCAGGCTT	1
CATCTCCTCCTCCCAGCTCCTCCCTAGTGCACAGATACCTATCGGCAGGCTC	1

c

African	Admixed American
East Asian	South Asian
Others	Non human primates

Fig. S1. Visualization of a VNTR in *SORL1*. (a) A visual display of the motif structure and composition of 110 alleles including 6 non-human primates (orangutan, gorilla, and chimpanzee) of a VNTR in SORL1 gene (chr11:121480539-121482037). (b) A list of detected motifs and their frequency. (c) Color codes for the super population depicted next to the sample name.

Decomposition algorithm

In the main manuscript, we introduced the tandem repeat (TR) decomposition problem, also known as the optimum parse problem. In this section, we provide a detailed description of the dynamic programming algorithm. Given an allele containing a TR sequence, a set of motif sequences M, and a scoring matrix, the objective is to identify the optimum parse of the TR allele using the input motifs. This will result in the decomposition of the sequence (see Features and Methods for the problem formulation).

We solve this problem using dynamic programming. Let $D[i, m, j]$ denote the maximum score aligning the prefix of the query sequence $x[1...i]$ to the input motifs, where the i-th character of the query sequence is matched to the j-th character of motif m. Then, the solution for the TR decomposition problem is:

$$\max_{m}\{D[|x|, m, |m|]\}, \tag{1}$$

where $|x|$ and $|m|$ denote the length of the query and motif sequence, respectively. The decomposed motifs can be obtained by tracing back the optimal path.

The recurrence for the dynamic programming can be calculated as follows. Let $S(i, j, m)$ denote the score when i-th character of query x is matched with j-th character of motif m, and S_{indel} denotes the score for insertion and deletion. Then, for $m \in M$, $1 \leq i \leq |x|$ and $1 \leq j \leq |m|$

$$D[i, m, j] = \begin{cases} \max \begin{cases} \max_{m' \in M}\{D[i-1, m', |m'|]\} + S(i, m, j) \\ D[i-1, m, 1] + S_{indel} \end{cases} & \text{if } j = 1 \\ \\ \max \begin{cases} D[i-1, m, j-1] + S(i, m, j) \\ D[i-1, m, j] + S_{indel} \\ D[i, m, j-1] + S_{indel} \end{cases} & \text{if } j \neq 1 \end{cases} \tag{2}$$

For initialization,

$$D[i, m, j] = \begin{cases} 0 & \text{if } i = 0 \text{ and } j = 0 \\ D[i, m, j-1] + S_{indel} & \text{if } i = 0 \text{ and } j \neq 0 \\ D[i-1, m, j] + S_{indel} & \text{if } i \neq 0 \text{ and } j = 0 \end{cases} \tag{3}$$

where $m \in M$, $0 \leq i \leq |x|$ and $0 \leq j \leq |m|$. The recurrence is similar to the Needleman–Wunsch algorithm (also known as global alignment algorithm), except when $j = 1$, where transitions from other motif spaces are allowed.

Customized scoring matrix for multiple sequence alignment

For each TR, we use a customized scoring matrix for aligning encoded sequences. Let k denote the number of distinct motifs (symbols). Then, we consider a k by k scoring matrix, where the (i, j) element in the matrix represents the score between the i-th symbol and j-th symbol, for $1 \leq i \leq k$ and $1 \leq j \leq k$. The diagonal values represent the scores between two identical symbols (match) and others for mismatches. For a match, we use a score of 2 by default, but for mismatches, we use two different mismatch scores. For pairs of symbols, where the edit distance between the motifs, which the symbols are referring to, is 1, we set the mismatch score to -1. Otherwise, we set the mismatch score to -2. For the pair of motifs, where the longer motif is $\geq$ 30 bp, we allow 1 more edit distance for every 30 bp. This scoring strategy helps to avoid too many gaps in the alignment.
